# Mid- to long-term effects of two different biological reconstruction techniques for the treatment of humerus osteosarcoma involving caput humeri

**DOI:** 10.1186/s12957-020-1797-z

**Published:** 2020-01-29

**Authors:** Weitao Yao, Qiqing Cai, Jiaqiang Wang, Jingyu Hou

**Affiliations:** Department of Bone and Soft Tumor, Affiliated Cancer Hospital of Zhengzhou University, Henan Cancer Hospital, Zhengzhou, Henan 45000 People’s Republic of China

**Keywords:** Proximal humerus, Osteosarcoma, Biological reconstruction, Clinical result, Complication

## Abstract

**Background:**

The proximal humerus is one of the most common sites of primary or metastatic malignant tumors. Reconstruction of the shoulder after tumor resection is controversial and challenging. When intra-articular resection is performed, biological reconstruction (osteoarticular allograft and autologous bone implantation) may be the first choice rather than prosthetic reconstruction.

**Objective:**

To observe the mid- to long-term effects of oncologic, reconstructive, and functional outcomes of two different biological reconstruction methods for resection of humerus osteosarcoma involving caput humeri.

**Methods:**

This was a retrospective study of 13 consecutive patients who underwent humeral reconstruction of osteosarcoma including caput humeri using osteoarticular allograft (*n* = 7) and tumor bone inactivated and reimplantation (TBIR, *n* = 6) in our clinic between 2007 and 2017. Patients’ general information, resection and reconstruction techniques, oncological and functional outcomes, and complications were collected and evaluated. Different complications of implantations were compared and analyzed for the different biological methods.

**Results:**

The study included ten males and three females with an average age of 19.15 years. The operation time was about 3.65 h with an average blood loss of 631 ml. The resection tumor bones were 13–45 cm (23.54 cm on average). The mean follow-up period was 5.27 years. The shoulder movement was 10–70° (average, 44.00°) in abduction, 0–30° (average, 14.17°) in flexion, and 0–20° (average, 11.90°) in extention at the last follow-up. The complications included fracture in four TBIR patients and two allograft patients with an average of 2.67 years postoperation. Fracture rate was higher and appeared time was earlier in TBIR patients than in allograft patients (*p* = 0.04); caput humeri absorption occurred in all seven allograft patients and three TBIR patients at an average of 3.10 years after surgery; severe graft bone resorption appeared in five TBIR patients and two allograft patients at an average of 2.57 years of follow-up.

**Conclusions:**

Humerus biological reconstruction involving caput humeri was associated with a high complication rate and acceptable limb function in the mid to long term. New combined biological methods should be explored and adopted in the future.

## Background

The proximal humerus is the third most common site of osteosarcoma and the second most common site of all bony sarcomas, with a predilection for metastatic disease [1–3]. Amputation of the upper limb is very mutilating, and artificial limbs provide limited function and poor cosmesis. With imaging modalities, careful performance of biopsy, neoadjuvant and adjuvant chemotherapy, and complete surgical resection of the tumor, limb-sparing surgery has become a reasonable alternative to amputation for humerus malignant tumors [1–5]. The most important aspect of limb-salvage surgery is to preserve elbow and hand function after excision of tumors of the proximal humerus, although the shoulder may only retain a limited active range of movement [6, 7].

Optimal methods for shoulder reconstruction after resection of the proximal humerus including caput humeri are controversial and challenging [2], especially in skeletally immature patients or massive bone resected, due to the narrow medullary canal, small length of the remaining bone, poor compliance in immobilization, and less soft tissue available for coverage [8, 9]. Currently, commonly used reconstructive options following caput humeri resection include preservation of the mobile glenohumeral joint using a prosthesis, osteoarticular allograft or allograft prosthesis composite, fibular or autoclaved humeral autograft, and the clavicula pro humero procedure [10–13]. The reconstruction method is often determined by factors such as the patients’ economic status, the tumor type and extension, the surgeon’s skills, and the availability of instruments [5].

When proximal humerus tumor does not invade the joint capsule or into the articular cavity, intra-articular resection can be performed. Surgeons can obtain a wide margin by preserving the proximity of the neurovascular bundle, deltoid muscle, sufficient deltoid, rotator cuff tendon, and part of the joint capsule to restore glenohumeral joint mobility, especially abduction [14, 15]. Of the many reconstructive procedures for treating proximal humerus involving caput humeri, the most commonly applied method is biological reconstruction (osteoarticular allograft and autologous bone implantation) rather than prosthetic reconstruction, especially in young patients or patients with a large bone defect after tumor resection [9]. Theoretically, biological reconstruction has merits such as creating bone stock for possible future revision, and the attachment of remaining deltoid muscle and rotator cuff tendons to the soft tissue of the graft provides better stability and active range of motion of the shoulder, which should lead to a better overall function and higher patient satisfaction. Yet, numerous studies have reported high rates of major complications including fracture, nonunion, subchondral collapse, and infection, which often require removal or revision of the osteoarticular graft [9–17]. Additionally, few proximal humerus allografts can match the small sub-acromial size in young children.

The purpose of this study was to observe the mid- to long-term oncologic, reconstructive, and functional outcomes of patients who underwent two biological reconstruction methods for humerus osteosarcoma involving caput humeri, namely osteoarticular allograft (OAA) and tumor bone inactivated and reimplantation (TBIR).

## Methods

### General information

**Fig. 1:**
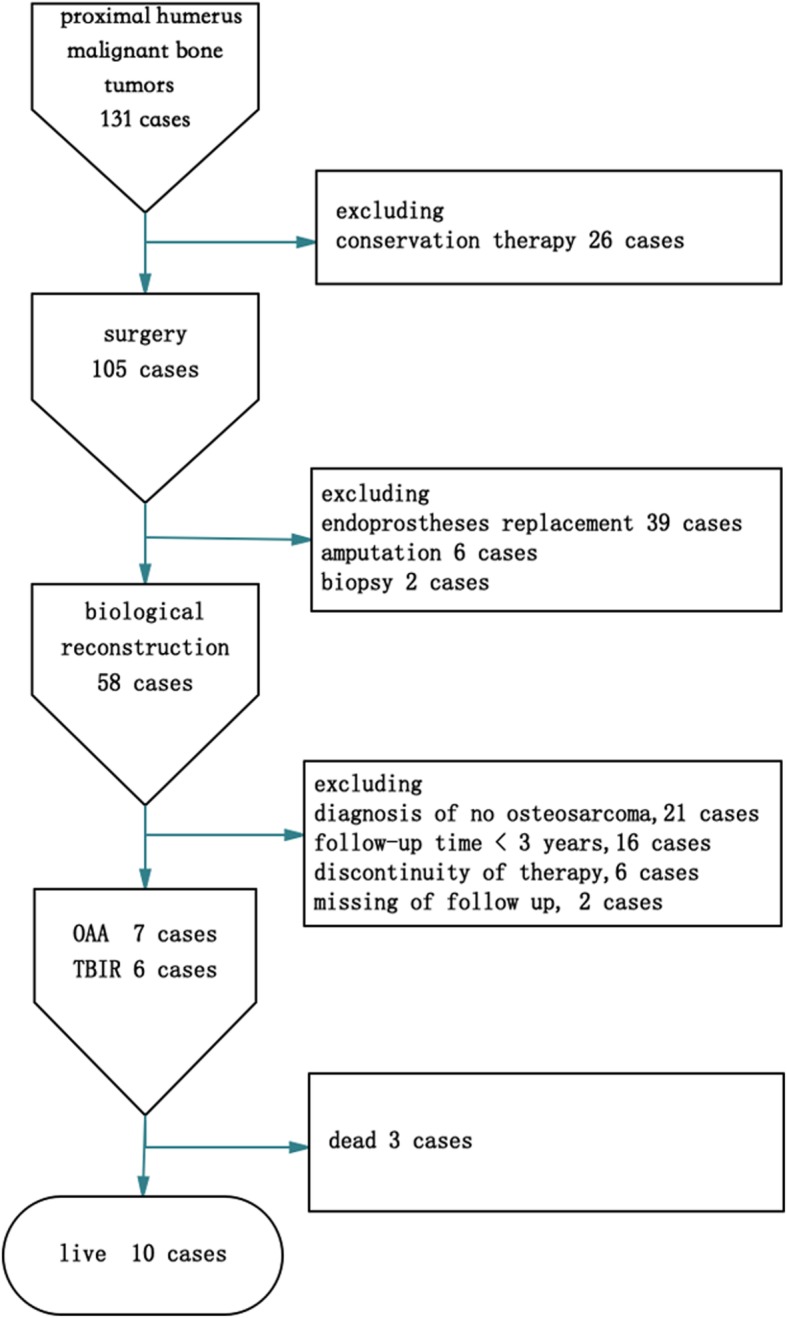
Sample decision tree illustrated the including and excluding procedure. There were 13 cases in the final selection

**Fig. 2:**
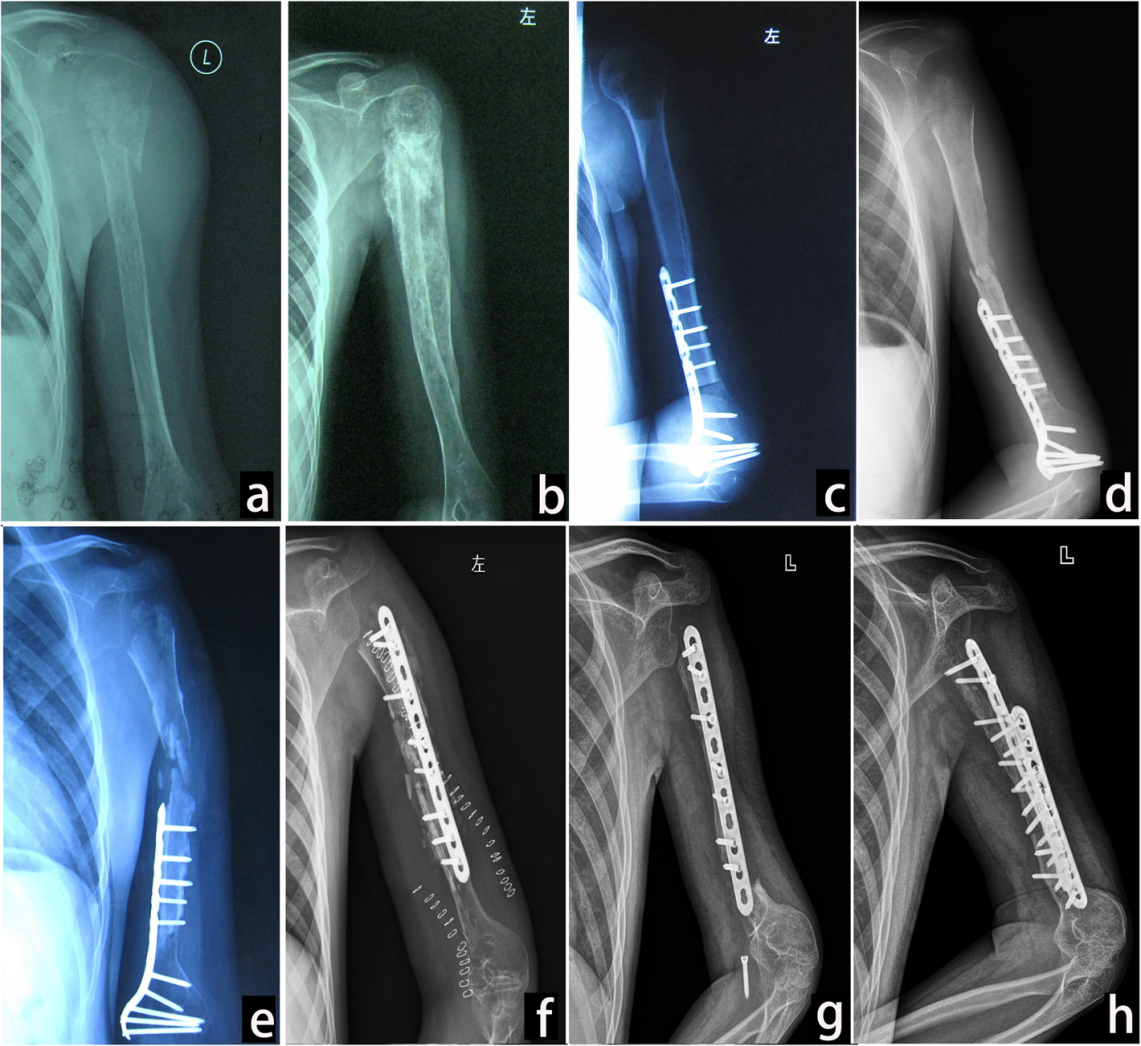
Osteoarticular allograft of a 12-year-old male patient (case 1). **a** Photograph at the first consultation. Pathological fracture was seen at metaphysis. **b** Photograph after two cycles of new adjuvant chemotherapy. New bone formation around the humerus. MSTS resection classification of S345E1. **c** Postoperation photograph of osteoarticular allograft. **d** Graft bone fracture and subchondral collapse after 2 years of follow-up. **e** Severe graft bone resorption (GBR) and caput humeri absorption (CHA) after 3 years of follow-up. **f** Change in fixation and fibular implantation after 4 years of follow-up. **g** Fracture at the graft-host junction with fixation loosens after 5 years of follow-up. **h** Bone healing after 8 years of follow-up by adding another fixation and bone implantation

**Fig. 3:**
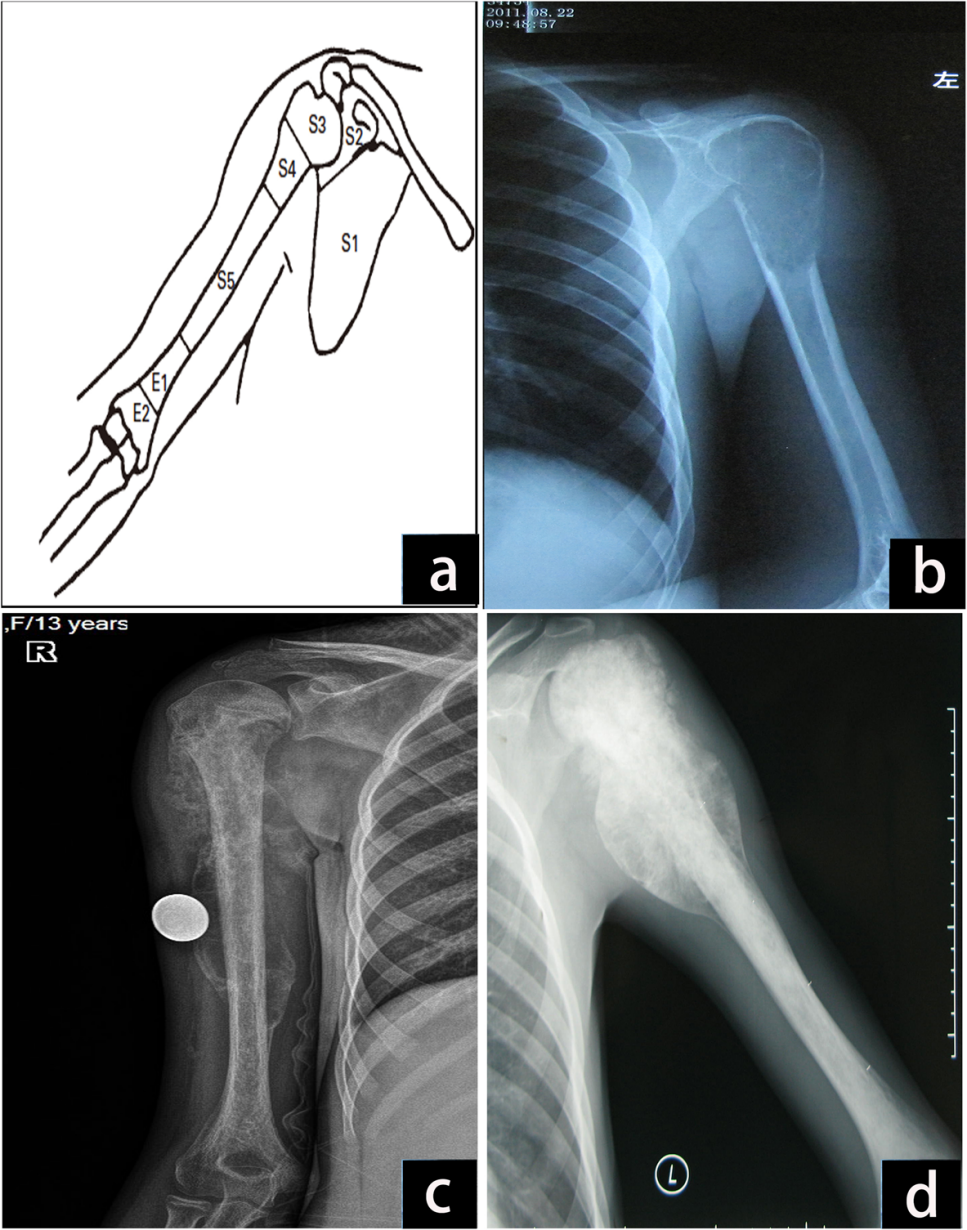
MSTS resection classification system for shoulder girdle and application. **a** MSTS resection classification system. **b** Defect after resection classification of S345 in case 2. **c** Defect after resection classification of S345E1 in case 1. **d** Defect after resection classification of S345E1E2 in case 4

There were 131 patients who had proximal humerus malignant bone tumors and presented to our ward between January 2007 and January 2017. We used the inclusion criteria of biological reconstruction including osteoarticular allograft or autograft for treatment of primary osteosarcoma of bone; absence of prior surgical treatments for the sarcoma; complete clinical, radiographic, and pathologic records; and minimum follow-up of 3 years from bone graft reconstruction. We also excluded patients who received discontinuity therapy and missed the follow-up. The final study group consisted of 13 consecutive patients with humerus osteosarcoma involving caput humeri (see Fig. 1). The study was approved by all patients and in accordance with the ethical guidelines of the Affiliated Cancer Hospital of Zheng Zhou University. All patients were evaluated with plain radiographs, magnetic resonance imaging, and computed tomography (CT) of the involved extremity, as well as total body bone scans and chest CT scans. Angiography was performed in certain patients to verify that the neurovascular bundle and rotator cuff were not involved. Tissue diagnosis was obtained through core needle biopsy of the lesions. All patients were diagnosed with osteosarcoma (11 stage IIB and 4 stage IIIA lesions). Three patients developed pathological fractures (Fig. 2a). After preoperative evaluation and neoadjuvant chemotherapy for osteosarcoma, all patients (7 cases) had intra-articular proximal humeral resection and osteoarticular allograft reconstruction, and 6 had TBIR (4 cases of tumor invasion and 2 cases of economic reasons). Soft tissues were resected to ensure negative margins with intraoperative biopsies. An effort was made to preserve as much of the abductor mechanism as possible in a safe margin. According to the Musculoskeletal Tumor Society (MSTS) resection classification system [18] (Fig. 3a), the defects were classified as S345 (9 patients; Fig. 3b), S345E1 (2 patients; Fig. 3c), and S345E1E2 (2 patients; Fig. 3d). The patients’ information is shown in Table 1.

**Table 1:** Characteristics and results of the 13 patients

Case	Gender/age (years)	Stage	MSTS resection system	Resection segment length (cm)	Reconstruction methods	Follow-up (years)	Patient status	Complications	Reoperations
1	M/12	IIB	S345E1	25	OAA	8	NED	CHA+GBR+fracture	Fixation+graft
2	M/23	IIB	S345	20	OAA	10	NED	CHA	None
3	M/25	IIB	S345	15	OAA	6	NED	CHA+nonunion	None
4	M/18	IIIA	S345E1E2	45	OAA	3	STD	CHA	Fixation+graft
5	F/13	IIIA	S345E1E2	41	TBIR	5	NED	GBR+fracture	Fixation+graft
6	M/30	IIIA	S345E1	33	TBIR	9	NED	CHA+fracture	Fixation+graft
7	F/24	IIB	S345	15	TBIR	4	NED	CHA+GBR+infection	Fixation+graft
8	M/16	IIB	S345	18	TBIR	4	NED	CHA+GBR nonunion	None
9	M/16	IIB	S345	16	TBIR	6	NED	GBR+fracture+nonunion	Prosthesis replacement
10	F/13	IIB	S345	18	OAA	3	STD	CHA	None
11	M/9	IIIA	S345	22	OAA	7	NED	CHA+GBR	Fixation+graft
12	M/34	IIB	S345	25	OAA	4	STD	CHA	None
13	M/16	IIB	S345	13	TBIR	3	NED	GBR+fracture	Fixation+graft

*TBIR* tumor bone inactivated and reimplantation, *OAA* osteoarticular allograft, *MSTS* Musculoskeletal Tumor Society, *TESS* Toronto Extremity Salvage Score, *CHA* caput humeri absorption, *GBR* graft bone resorption, *NED* none evidence of disease, *STD* succumbed to disease

### Surgical technique

All patients underwent resection using a deltopectoral approach that included the biopsy scar, extending as far distally as required for adequate margins. Two patients with S345E1E2 had total humerus resection. All the surgeries were carried out by senior doctors.

**Fig. 4:**
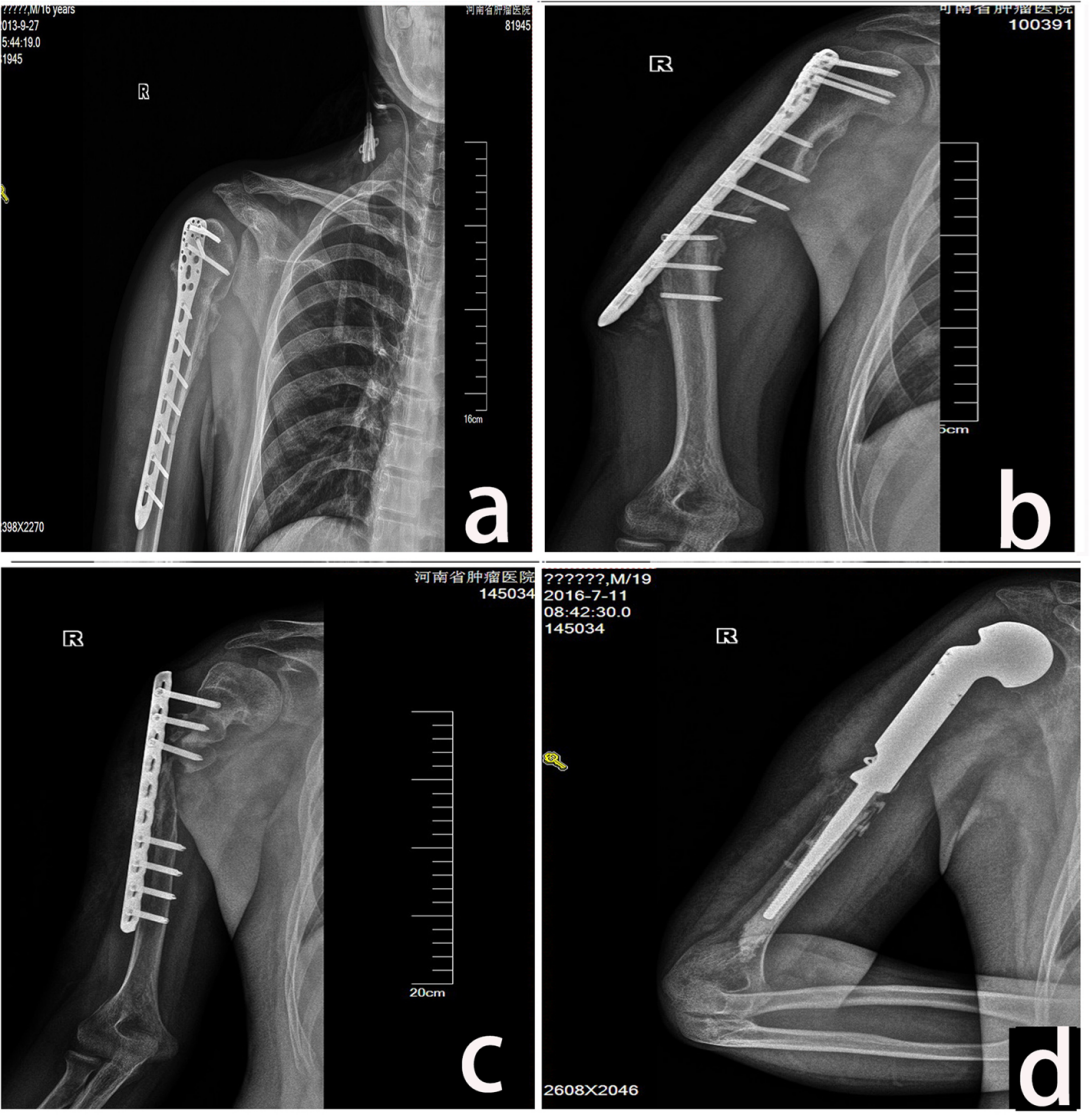
TBIR in case 9. **a** Photograph 2 weeks after TBIR operation in a 16-year-old patient. **b** Fracture with bone resorption after 1 year of follow-up. **c** Change in fixation and autologous bone implantation after 2 years of follow-up. Bone resorption with nonunion continued, and fixation was loosened. **d** Prosthesis changes after 3 years of follow-up

During surgery, the deltoid attached to the acromion and inserted to the proximal humerus was sparied as a myocutaneous flap with soft tissue. The rotator cuff was divided approximately 1 cm off from insertion onto the proximal humerus. The axillary nerve was sacrificed or preserved in malignant disease on a case-by-case basis according to its proximity to the bone. In total humerus resection patients, humeroulnar and humeroradial joint capsule was resected, and the distal humerus was disarticulated. After resection of the tumor, a size-matched proximal humeral osteoarticular allograft was inserted in seven patients (Fig. 2c) and tumor bone reimplantation after inactivation in six patients (Fig. 4a). In inactivated bone patients, the tumorous cortex and medullary cavity were separated from the rest of the proximal humerus. Then, the humerus was fixed with interlocked bent plates that followed the shape of the residual humerus. The bone was placed in anhydrous alcohol for 40 min and then washed with a large amount of physiological saline. The allografts were stored fresh-frozen and non-irradiated. The allograft or autologous osteoarticular graft was attached to the patient’s residual humerus with a lateral dynamic compression plate and screws. Autograft from the iliac crest was placed around the osteotomy site in 10 of the 13 cases. Total inactivated and allograft humerus was applied in 1 patient each without internal fixation. Shoulder stability was achieved using the remaining capsule and rotator cuff musculature sutured to the labrum and around the humeral head of the graft, and the pectoralis major and latissimus dorsi muscles were reattached to their anatomic insertion sites on the allograft. The distal humerus joint was treated by reduction, and the capsular was also repaired. Immediate postoperative immobilization was applied in all cases with either casts or splints for a minimum of 6 weeks.

### Postoperative management and follow-up

After the surgical procedure, patients were immobilized in a thoracobrachial cast or orthosis with 95° abduction and 15° shoulder anterior position. All patients were started on early gentle range of motion (ROM) exercises of the elbow (except for total humerus graft), wrist, and hand. The average duration of immobilization in a thoracobrachial cast was 6 weeks. After removal of the cast or brace, the arm was supported in a sling for an additional 1–3 months. At the same time, patients began active ROM exercises and physiotherapy. All patients received additional adjuvant chemotherapy and routine follow-up every 3 months for the first 2 years, every 6 months for the following 2 years, and every 12 months thereafter. The involved shoulder joint functional results were described according to the physical examination findings including abduction, flexion, and extension before and after surgery. Radiographs and magnetic resonance imaging of the operated limb and CT scans of lung were made at routine intervals.

### Adverse events and statistical analysis

We defined various failure modes as events. An event constituted unsuccessful biological reconstruction including common complications such as fracture, nonunion, bone resorption, infection, hardware failure, graft removal, or amputation. For radiographic evaluation, fracture was defined as discontinuity of the graft bone; caput humeri absorption (CHA), as partial or complete humerus head resorption in the epiphyseal region; graft bone resorption (GBR), cortical, or total graft bone loss of more than 3 cm without new bone formation; and nonunion, no evidence of radiographic bridging of the approximated ends between the graft and host bone at least 2 months apart at a minimum of 6 months after the operation. Complication in different reconstruction methods was compared using the nonparametric Mann-Whitney test. *p* < 0.05 was considered to indicate a statistically significant difference. SPSS software (version 11.5; SPSS, Inc., Chicago, IL, USA) was used. All the data collection and processing were carried out by junior doctors.

## Results

### Oncological outcome

There were 10 males and 3 females who underwent biological reconstruction of the proximal humerus, and their average age at surgery was 19.15 (9–34) years. The operation time was 3.65 (2.5–5) h, and the blood loss was 631 (400–1000) ml. The mean follow-up period was 5.27 (3–10) years. The average resection length was 23.54 cm (range, 13–45 cm) in 13 cases. Distant metastases occurred in 4 cases, of which 1 was treated by resection of the lung metastatic lesions with no evidence of disease. Three other patients had multiple lung nodes; they received second-line chemotherapy and died 1 year later. Thus, there were 10 patients with disease-free survival at the last follow-up.

### Complications (see Table 2)

**Fig. 5:**
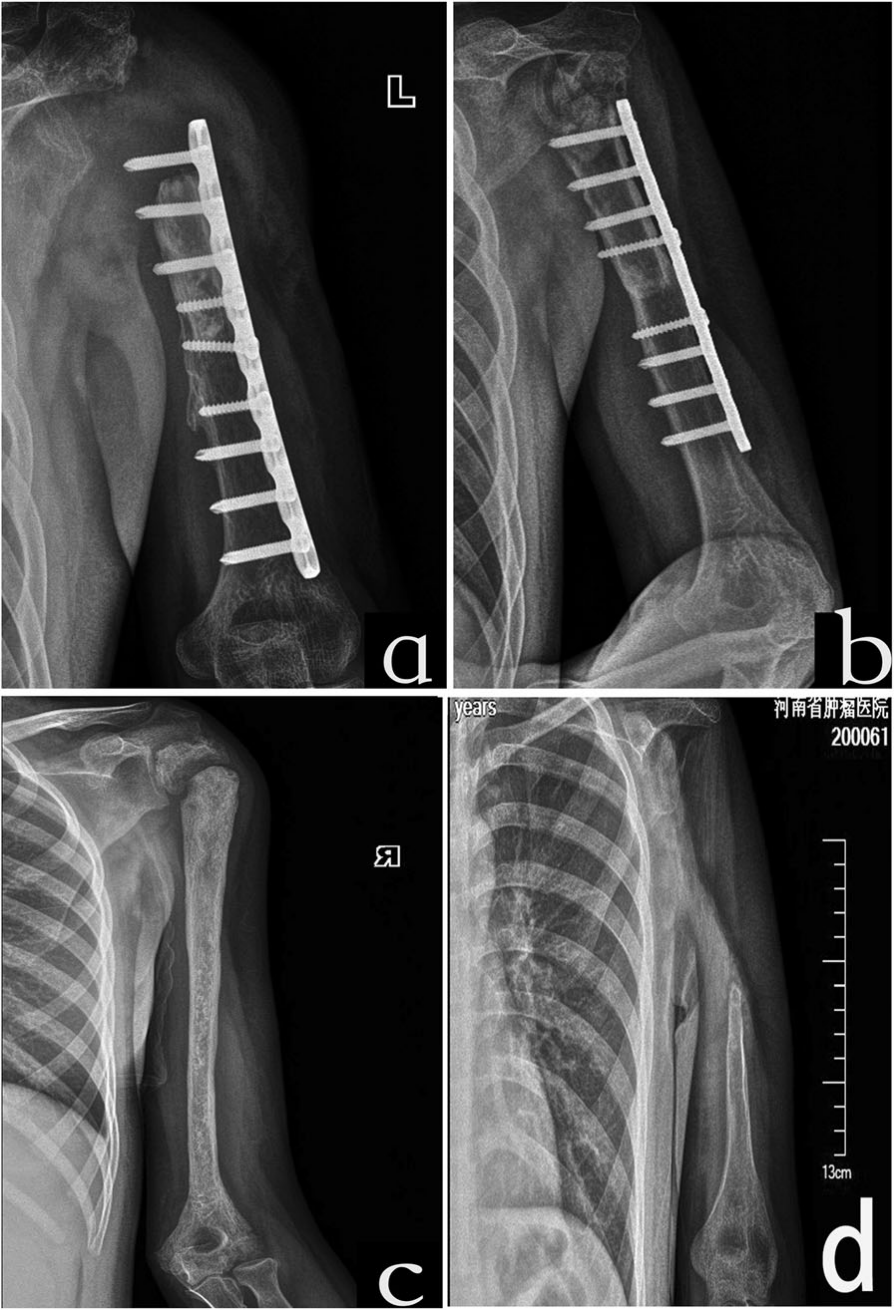
Absorption of humeral head. **a** Photograph at 10 years of follow-up in case 2; allograft caput humeri absorbed completely in a 23-year-old patient. **b** Photograph at 4 years of follow-up in case 12; allograft humeral head absorbed partly in a 34-year-old patient. **c** Photograph at 5 years of follow-up in case 5; graft bone resorption and metaphysis fracture happened in a 13-year-old patient in TBIR treatment. **d** Photograph at 4 years of follow-up in case 7; graft bone completely resorbed in a 24-year-old patient in TBIR treatment

Fracture occurred in six patients including four (57.14%) in the TBIR (Fig. 4b) and two (33.33%) in the allograft (Fig. 2d) at an average of 2.67 (1–7) years of follow-up. Fracture rate was higher and appeared time was earlier in TBIR patients than in allograft patients (*p* = 0.04). The location was at the metaphysis in three and at the diaphysis in three. The metaphyseal fractures occurred without the protection of plate in two cases. Fracture appeared at the distal of autograft host junction in one patient after removal of fixation (Fig. 2g). Fractures accompanied by fixation loosened and broken in two patients (Fig. 2g and 4b), accompanied by subchondral collapse and caput humeri absorption in two patients (Fig. 2d), and accompanied by nonunion in one patient. Fracture was corrected by fixation changes and iliac bone implantation in three patients and healed in the late follow-up (Fig. 2h). The fracture bone with fixation failure was substituted by tumor prosthesis in one patient (Fig. 4d). Two patients with metaphysis fracture had no fixation protection and untreated without pain (Fig. 5c). Caput humeri absorption is the most common complication in osteoarticular allograft replacement. In this study, the complication of caput humeri absorption occurred in all seven patients (100%, Fig. 5a, b). Caput humeri was absorbed in three of six (50%) TBIR patients. There were no differences in the two reconstruction methods (*p* = 0.50). The caput humeri absorption happened at an average of 3.10 (1–5) years after the operation. All patients had no pain or shoulder subluxation with limited movement of shoulder joint, especially abduction, and received nonoperative treatment. Severe graft bone resorption (GBR) was another complication in auto/allograft, which occurred after an average of 2.57 (1–5) years of follow-up in seven (53.85%) patients (five of six TBIR patients, two of seven allograft patients, see Figs. 2e and 4c). The graft was repaired by autologous non-vascularized fibular in two patients, and removed and replaced by prosthesis or segment cemented spacer in two patients, and three patients remained untreated. One patient with allograft and two TBIR patients had radiographic nonunion. The patient underwent revision surgery with bone grafting, even though nonunion persisted without pain. Union was achieved after this second procedure. One patient (case 7) with OAA and severe GBA had deep infection and sinus formation. The wound healed after graft bone was taken out and debrided (Fig. 5d). There was no disease recurrence in all 15 patients, and no patients with surgical complications accepted or received an amputation procedure.

**Table 2:** Complications in the two construction methods

Complications	OAA		TBIR		*p* value
	Cases	Rate (%)	Cases	Rate (%)	
Fracture	2	28.57	4	66.67	0.04
CHA	7	100.00	3	50.00	0.50
GBR	2	28.57	5	83.33	0.09
Nonunion	1	14.29	2	33.33	1.00

*CHA* caput humeri absorption, *GBR* graft bone resorption

### Functional outcome

The length of bone that was resected influenced the functional outcome. None of the 15 patients was able to abduct their shoulder more than 90°. The shoulder movement was 40–120° (average, 75.31°) in abduction, 20–70° (average, 38.00°) in flexion, and 10–40° (average, 21.77°) in extension before surgery, and 10–70° (average, 44.00°) in abduction, 0–30° (average, 14.17°) in flexion, and 0–20° (average, 11.90°) in extension at the last follow-up in these patients with no difference in the two reconstruction methods.

## Discussion

Malignant tumors of the proximal humerus are challenging problems, and numerous reconstructive methods have been described [2–17, 19, 20]. In 1990, Gebhardt et al. [21] first reported their experience with osteoarticular allografts in 20 patients. Approximately 70% of their patients had minimum pain and returned to normal activity with a high complication rate, including three infections, seven fractures, one nonunion, and one case of significant instability. Manfrini et al. [9] compared their biological, endoprosthetic reconstruction with amputation methods in proximal humerus in children in a single institute and commanded biological reconstruction methods in these special patients. van de Sande et al. [22] found that the endoprosthetic group had the smallest complication rate of 21% (*n* = 1), compared to 40% (*n* = 4) in the allograft prosthesis composite and 62% (*n* = 8) in the osteoarticular allograft group after caput humeri resection. The authors concluded that endoprosthesis should be the first choice in keeping the functional results and glenohumeral stability, but their patients have primary benign or malignant tumor or metastatic disease at an average of 44.8 (16**–**83) years. The defect length of proximal humerus was not clear.

In our study, the patients were 19.15 (9–34) years old with a bone defect of 23.54 (13–45) cm. Residual humerus in E1 and E2 region or children were not suitable for endoprosthetic reconstruction because of the small length of remaining bone and narrow medullary canal. There was an extremely high rate of complications including fracture, especially at the metaphyseal graft; caput humeri absorption; severe resorption of the graft; and nonunion. We observed caput humeri absorption in all allograft patients (100%) in 5 years. Higher rate of severe graft resorption and fracture happened earlier in TBIR patients than in OAA patients. Unlike bone graft failure in the lower limb, patients with severe complications, even though all the bone graft was absorbed (see Fig. 5d), could not accept shoulder joint amputation.

We did not find the ideal shoulder joint movement, especially abduction. The reasons may be that in most or all of our patients with high-grade sarcoma, the axillary nerve could not be preserved because of tumor extension. Most patients experienced one or more major complications such as caput humeri absorption or fracture; thus, the abductor mechanism was disrupted in almost all of these patients. Although excellent long-term outcomes were rare in our series, a flail shoulder is often the best alternative for the patient who does not need to use the hand in space; the same was reported by other authors [7–17, 23]. We also find that functions of the elbow, wrist, and hand remained in a flail limb and can meet the requirements of daily life with the help of an orthotic or the contralateral hand.

Fracture is one of the main complications in osteoarticular allografts or autologous bone implantation. The reason may be that the grafts incorporate into host bone by creeping substitution, which involves partial reabsorption of the bone graft and formation of new, vascularized bone, leaving the graft bone in a weakened state during the process [16, 17]. Factors that have been associated with an increased risk of fracture in our study include nonunion, the combination of chemotherapy, and unilateral plate fixation. To reduce the fracture rate, some authors recommend minimizing the number of screws or using intramedullary methods of allograft fixation because screw holes create stress risers where fractures can occur [17]. Jamshidi et al. [24] described a reduced rate of fractures in cement-filled allografts of the proximal humerus. They also reported decreased severity of subchondral fractures and subsequent articular collapse in cement-filled allografts [25]. We found that a fracture occurred after 4 years of follow-up with bone healing and removal of fixation in one patient. This indicated that the bone healing could not reach the normal strength and structure of the normal bone. Metaphyseal graft fracture is a well-known problem of osteoarticular grafts. These fractures caused substantial collapse of the articular surface and caput humeri absorption. Fractures occurred at an average of 1.45 (0.2–5) years postoperatively. No fractures occurred after 60 months of follow-up. Factors associated with fracture may involve movement of shoulder joint in early time, less protection of internal fixation, less blood supply of the graft, and joint fluid corrosion.

Severe GBR is another main complication in massive bone implantation. The resorption happened in five of six TBIR patients and two of seven allograft patients with an average of 2.57 (1–5) years postoperatively. As with fracture, bone resorption is related to problems with the incorporation of the dead massive graft bone into the living host bone. If the living host bone has poor osteoinductive capacity or insufficient fixation or blood supply of the graft bone, the bone resorption may occur rather than bone formation. The risk may be reduced by achieving rigid, long, and stable fixation and combining with vascularized autologous bone graft [25, 26]. The resolved methods are using cancellous autograft at junctions and change fixation in this series.

Although much more severe complications happened in our study, biological construction such as OAA and TBIR remains a viable option for limb-sparing tumor resections in certain patients with tumor involved in most of S5 or E1 and E2 area or with skeletally immature skeleton. Endoprostheses are not always available in these patients for residue and small medullary canal fixation and big intramedullary stems. Moreover, the cortical resorption at the implant-bone interface, both in cemented and uncemented stems, influences the amount of bone stock available for future revisions in long-term survivor patients [19].

The limitation of this study includes the retrospective character, the inevitable small number of patients, the limited following time, the selection bias of patients, and the operation methods. Further study should be taken in this area, such as combined allograft and vascularized fibula graft, TBIR, or allograft with prosthesis and fixation, in promoting the clinical effect and survival rate of the biological construction in proximal humerus.

## Conclusions

Humerus biological reconstruction including caput humeri was associated with a high complication rate and acceptable limb function in the mid to long term. New combined biological methods should be explored and adopted in the future.

## Data Availability

The datasets used during the current study are available from the corresponding author on reasonable request.

## Abbreviations

CHA: Caput humeri absorption
CT: Computed tomography
GBR: Graft bone resorption
MSTS: Musculoskeletal Tumor Society
NED: None evidence of disease
OAA: Osteoarticular allograft
OS: Overall survival
ROM: Range of motion
STD: Succumbed to disease
TBIR: Tumor bone inactivated and reimplantation
TESS: Toronto Extremity Salvage Score
